# Dipy, a library for the analysis of diffusion MRI data

**DOI:** 10.3389/fninf.2014.00008

**Published:** 2014-02-21

**Authors:** Eleftherios Garyfallidis, Matthew Brett, Bagrat Amirbekian, Ariel Rokem, Stefan van der Walt, Maxime Descoteaux, Ian Nimmo-Smith, Dipy Contributors

**Affiliations:** ^1^Computer Science Department, University of SherbrookeSherbrooke, QC, Canada; ^2^MRC Cognition and Brain Sciences Unit, University of CambridgeCambridge, UK; ^3^Henry H. Wheeler, Jr. Brain Imaging Center, University of CaliforniaBerkeley, CA, USA; ^4^Department of Neurology and Graduate Group in Bioengineering, University of CaliforniaSan Francisco, CA, USA; ^5^Department of Psychology, Stanford UniversityStanford, CA, USA; ^6^Department of Mathematical Sciences, Division of Applied Mathematics, Stellenbosch UniversityStellenbosch, South Africa; ^7^http://dipy.org/developers.html

**Keywords:** diffusion MRI, DTI, DSI, HARDI, dMRI, Python, free open source software, tractography

## Abstract

Diffusion Imaging in Python (Dipy) is a free and open source software project for the analysis of data from diffusion magnetic resonance imaging (dMRI) experiments. dMRI is an application of MRI that can be used to measure structural features of brain white matter. Many methods have been developed to use dMRI data to model the local configuration of white matter nerve fiber bundles and infer the trajectory of bundles connecting different parts of the brain. Dipy gathers implementations of many different methods in dMRI, including: diffusion signal pre-processing; reconstruction of diffusion distributions in individual voxels; fiber tractography and fiber track post-processing, analysis and visualization. Dipy aims to provide transparent implementations for all the different steps of dMRI analysis with a uniform programming interface. We have implemented classical signal reconstruction techniques, such as the diffusion tensor model and deterministic fiber tractography. In addition, cutting edge novel reconstruction techniques are implemented, such as constrained spherical deconvolution and diffusion spectrum imaging (DSI) with deconvolution, as well as methods for probabilistic tracking and original methods for tractography clustering. Many additional utility functions are provided to calculate various statistics, informative visualizations, as well as file-handling routines to assist in the development and use of novel techniques. In contrast to many other scientific software projects, Dipy is not being developed by a single research group. Rather, it is an open project that encourages contributions from any scientist/developer through GitHub and open discussions on the project mailing list. Consequently, Dipy today has an international team of contributors, spanning seven different academic institutions in five countries and three continents, which is still growing.

## 1. Introduction

**Figure 1:**
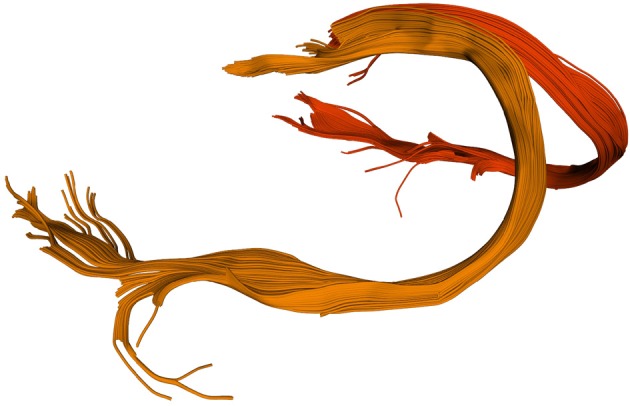
**The fornix is a C-shaped bundle connecting the hippocampus with the hypothalamus.** The body of the fornix divides into two legs knows as the crus of the fornix. We can see here a detailed recreation of the fornix using dMRI data processed with Dipy.

*Diffusion MRI* (dMRI) (LeBihan and Breton, 1985; Merboldt et al., 1985; Taylor and Bushell, 1985) is an MRI technique (Callaghan, 1991) that provides information about the structure of neuronal pathways found in the white matter and other body tissue with fiber-like structure (see Figure 1). dMRI acquires one or more *T*_2_ reference images, and a collection of diffusion-weighted images, in which *T*_2_ signal is attenuated according to the diffusivity of water along prescribed gradient directions (Behrens and Johansen-Berg, 2009; Jones, 2010). Because diffusion is hindered across nerve fiber membranes and less hindered along the length of nerve fibers, the signal is relatively more attenuated when diffusion-weighting is applied along the length of the fiber. Hence, the local structure of the neural tissue can be inferred from the measurements. This has led to many applications of the method, including diagnostic tools to assess the disruption of the microstructure and methods of tractography, which estimate the trajectories of nerve fibers that communicate information between different parts of the brain.

Because of its unique capability to characterize the microstructure of neural tissue, and the inferences that can be made using this information about structural connectivity, dMRI has had increasing popularity, with more than 5000 papers published in 2012 (according to PubMed). This popularity is also evident from the large number of software tools available for the analysis of diffusion-weighted images. Many of these tools are written in C/C++: 3D Slicer (Pieper et al., 2006), AFNI (Cox, 2012), MITK (Fritzsche et al., 2012), BrainVoyager QX (Goebel, 2012), DTI-Query/Quench (Sherbondy et al., 2005), FreeSurfer (Fischl, 2012), FSL-FDT (Smith et al., 2004), MedInria (Toussaint et al., 2007), MRtrix (Tournier et al., 2012), Diffusion Toolkit/Trackvis (Wang et al., 2007), FiberNavigator (Vaillancourt et al., 2011; Chamberland and Descoteaux, 2013). A few are written in other languages, such as R: TractoR (Clayden et al., 2011), Java: Camino (Cook et al., 2006) and Matlab: ExploreDTI (Leemans et al., 2009), AFQ (Yeatman et al., 2012) and others.

Dipy (*Diffusion Imaging in Python*) (Garyfallidis et al., 2011) is the first collective effort to create an open-source diffusion MRI analysis library using the Python language. Python is a general purpose, object-oriented programming language which was designed with an emphasis on code readability. This emphasis allows scientists who are not trained as software engineers to understand the computational steps taken during the analysis and to extend the software easily. Being an interpreted language, Python does not require additional compilation and linking, so scripts and libraries written only in Python are relatively easy to install and share. Python can be used interactively making it a good match for exploratory data analysis and methods development. Taken together, these properties of the language are powerful assets for the design of the next generation of medical imaging analysis tools.

In the past, we have found that many researchers used the available tools without necessarily understanding the underlying details, often because these were hidden from the users. Dipy tackles this problem in part by being free, open source (BSD license), simple and well documented. The environment for Python packages in imaging is healthy. There has been large growth in the number of Python users, there are many Python tools for scientific computing (Pérez and Granger, 2007; Pérez et al., 2011; McKinney, 2012), and there are complementary neuroimaging packages in Python^1^.

Dipy takes full advantage of the growing ecosystem of tools written for scientific computing in Python, and is built on top of production-ready, high-performance Python libraries. Primarily, Dipy depends on Numpy^2^. The core structure of this library is an implementation of an N-dimensional array class (van der Walt et al., 2011). Numpy arrays are used for representing numerical data in Python and enable efficient numerical computations through the use of vectorized operations, by avoiding data copying, and by minimizing the number of operations performed. Numpy is also used for matrix, tensor and linear algebra operations. Dipy further depends on Scipy^3^ for non-linear optimization and other volumetric operations. We use Cython^4^ in rare cases when both standard Python and Numpy/Scipy are not fast enough for the task at hand. Cython converts Python code into Python C extensions by interpreting static type declarations. The last required dependency for Dipy is Nibabel^5^. Nibabel is a package for loading and saving medical imaging file formats.

Dipy uses other optional libraries for visualization, testing and documentation. We use Matplotlib^6^ for 2D and 3D plotting and Python-VTK^7^ for more advanced 3D interactive visualization. Dipy uses nose^8^ for unit testing and Sphinx^9^ for automated documentation. Finally, we recommend using IPython^10^ as an interactive Python shell for calling and debugging scripts.

In the following sections, we explain the philosophy and main design concepts behind Dipy. We also give examples which cover different parts of the diffusion MR analysis pipeline from the analysis of diffusion signal in individual voxels to streamline generation by tractography algorithms and visualization of these streamlines.

## 2. Philosophy and mission

The purpose of Dipy is to make it easier to do better diffusion MR imaging research. We aim to build software that is clearly written, well explained and thoroughly tested, while being a good fit for the underlying ideas and providing a natural meeting point for collaboration.

We designed Dipy to be an international project that welcomes contributions from anywhere in the world. As for many other projects, we suffer from the tension between our desire to encourage new code and our need to keep Dipy well tested and well maintained, so that we and others can continue to use it and use it to build new things. To keep code quality high and help each other understand the new code, we use public code review. Each contribution, from any author, first gets proposed on the public website^11^. The code can be merged after everyone has had a chance to comment and ask questions. The same system allows the code to be tested automatically for test errors.

We believe that this discipline makes the project more attractive for new developers, because it is clear how decisions are made, and that each developer can and must interact as a peer with his or her colleagues on the project.

We are glad to see that Dipy has attracted an international and multi-departmental team of contributors from different levels of education (Master students, PhD students, Post-Docs, and Professors) spanning the fields of Computer Science, Medicine, Applied Mathematics, Biomedical Engineering and Psychology.

## 3. Terminology

As in most scientific fields, dMRI uses domain-specific terminology to describe the constructs of the measurement, as well as to present the interpretation of the results of the analysis. We rely on a recent paper (Côté et al., 2013), that proposes specific terminology for describing different constructs of the dMRI field. In this section we describe terms for concepts in the measurement and the results of the analysis. In subsequent sections, we use this terminology to explain the analysis code and interpretation of data.

The measurement in dMRI relies on the application of a pulsed magnetic gradient to make the measurement sensitive to diffusion. The degree of sensitization depends on a number of parameters, including the duration of the gradient, the time that elapses between pulses of the gradient, and the gradient amplitude. These parameters are together summarized in what is referred to as the *b-value*. As described above, the measurement is conducted with the magnetic field gradient applied in several different directions, and these are encoded in so-called *b-vectors*. These are unit vectors that describe the direction of the gradient relative to the scanner coordinate frame in which the gradients are applied. Different algorithms are used to determine the placement of these *b*-vectors (e.g., Jones et al., 1999; Caruyer et al., 2013).

While we are ultimately interested in the identification of the trajectories of bundles of axons, which are the long fiber-like part of a nerve cell along which electrical impulses are conducted from cell to cell (and whose size is on the μm scale), the measurement is conducted on a much larger scale. Typically, the measurement is conducted on a grid of *voxels* of approximately 2 × 2 × 2 mm. The scale of measurement limits us to describing the trajectories of *fascicles* of nerves, which are relatively large (mm–cm scale) bundles of axons traveling through the white matter together. One of the major achievements of this field is that it is now possible to reliably and accurately identify major fascicles in individual experimental participants or patients (Mori et al., 2005). These major fascicles are also known as *tracts*. This is a term taken from neuroanatomy and describes a group of neuronal axons within the central nervous system (mm scale).

For the purpose of interpretation of these data, a *fiber* can be any long and thin structure. Hence, fiber tracking is a general term that can be used in any field that reconstructs fibrous structures, such as white matter axon fibers, muscle fibers, prostate fibers and even celery fibers (Numano et al., 2006). *Fiber bundles* denote groups of fibers usually with an anatomical or functional meaning. These can be major tracts in the brain. Examples of major tracts are the arcuate fasciculus, which connects parts of the posterior temporal lobe with the frontal lobe; and the fornix (see Figure 1), which connects the medial temporal lobe with sub-cortical structures, such as the hypothalamus and amygdala. A fiber bundle in brain anatomy is synonymous to a tract, also often called fiber tract. The term tract can be misleading when talking for example about the corticospinal tract, because the corticospinal tract is in fact not a single tract but a group of tracts (including corticobulbar projections, the pyramidal tract, etc.).

*Tractography* is the computational process through which the fibers are detected and delineated. Tractography relies on the assumption that diffusion of water, as reflected in the dMRI measurement, occurs more freely along the axis of an axon, than across the membranes of the axon. Tractography is therefore usually done by finding the directions of diffusion in each voxel (see section 6) and stepping through the brain volume along the most likely directions of diffusion estimated in each location. This process generates so-called *streamlines*, which are imaginary lines that approximate the underlying fibers. Streamlines are also sometimes referred to as *tracks*. These are not to be confused with *tracts*: while a tract is a physical object, a track is a computational construct that only approximates the underlying fascicle or bundle of fibers. Confusingly enough *streamline bundles*, or simply *bundles* are often used to refer to a group of streamlines with similar shape and spatial characteristics (see section 8.1). These do not necessarily correspond to individual physical fiber bundles but are instead computational constructs that approximate the underlying anatomy.

## 4. General design aspects

**Figure 2:**
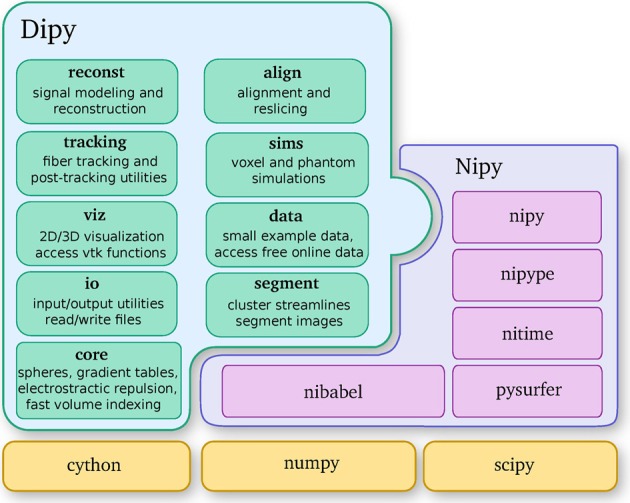
**Dipy is one of the main projects of the Nipy community and depends strongly on Numpy, Scipy, and Cython.** This diagram further shows the major Dipy sub-modules.

Dipy is built on the Scipy tool stack, which includes packages such as Numpy (numerical arrays and array computation), Scipy (scientific libraries, e.g., optimization, triangulation, special functions, and more), and Cython (an optimized Python to C compiler, used to achieve C-level code performance). Furthermore, Dipy is one of the major components of the Nipy (Neuroimaging in Python) ecosystem of medical imaging software (see Figure 2). Dipy also interacts effortlessly with other Nipy projects. For example, Nipype (Gorgolewski et al., 2011) has Dipy interfaces which allow building scalable dMRI workflows.

Dipy's API is designed to be intuitive, simple to use, and well documented. This allows researchers to build fairly simple Python analysis scripts that can build complex computational experiments while still being easy to read. In addition, since the code is entirely free and open, Dipy is ideally suited to facilitate reproducible research (Donoho, 2010).

Like many modern open source projects, Dipy is hosted on GitHub—an online repository that hosts open-source software, using the Git source-code management system to perform revision control. Any contributor with a (free) GitHub account can propose changes via a Pull Request (PR). These PRs have an interface that allows all interested coders to discuss the code and iterate on it before being we make the final decision to include the code in dipy. The discussions also allow for line-by-line comments; these can be useful for sharing improvements in the code or documentation, and learning new techniques from more experienced developers.

To assist developers, Dipy uses the Travis continuous integration system. Any time a new PR is proposed, Travis receives an automated signal to commission a new virtual machine to run the tests. A file in the Dipy repository tells Travis how to install the necessary dependencies, build a clean copy of Dipy, and run Dipy's test suite against the new changes. Travis integrates with GitHub to post the results back to the PR interface. This means that we can check any new code for errors, and make sure that code included into Dipy passes all the tests. After the code reaches the main development branch of Dipy, we run more comprehensive tests on all our supported platforms using a buildbot^12^ system hosted at nipy.bic.berkeley.edu. This gives us early warning of any problems on platforms such as Windows or OSX. Dipy also follows a test-driven development philosophy, whereby all code should be accompanied by a suite of tests to exercise all corner cases (Maximilien and Williams, 2013).

Dipy uses Sphinx to build the project documentation. Sphinx is the documentation system used by the main Python language project and many other Python projects, including all the neuroimaging projects. It takes plain-text ReStructuredText as input and converts it to either HTML, LaTeX or PDF formats. Sphinx takes API documentation directly from the docstrings in the source code, so that the API documentation does not get out of date with the code.

Dipy's main sub-modules (see Figure 2) are *core*, *reconst, tracking, viz, io, align, data, sims* and *segment*. *Core* contains general functions that can be used in any other sub-module. *Reconst* contains classes for estimating diffusion metrics in individual voxels. *Tracking* holds classes for fiber tracking and streamline processing. *Viz* is used for 3D visualization and interaction. *Io* offers input/output utilities when they are not available in Nibabel. *Align* provides tools for alignment and reslicing of volumes or streamlines. *Sims* is focused on creating synthetic simulations. *Data* is used for downloading public datasets. *Segment* concentrates on segmentation of images and clustering of streamlines.

In this paper we use code listings to illustrate the use of Dipy. For example, the following code snippet shows how to find your current version of Dipy.


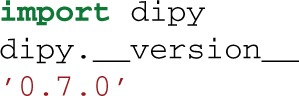


## 5. Pre-processing

### 5.1. Load/save data

The most basic operation that we perform in neuroimaging is to load files containing data that have been generated from an MRI scanner. Surprisingly this is often a difficult task as different scanners and software read/write the data in different ways. Fortunately Nibabel, a library for reading medical imaging formats, provides support for ANALYZE (plain, SPM99, SPM2), GIFTI, NIfTI1, MINC, MGH, ECAT, PAR/REC, Freesurfer (geometry, morphometry files), and with a growing support for DICOM.

The most common file format used in dMRI is the NIfTI1 format. Assuming that we have a file with our 4D raw diffusion data we can load it in the following way:


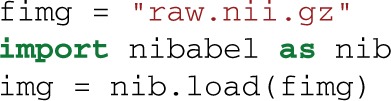


Where fimg holds the filename for the 4D NIfTI1 file, nib is a shortcut for the Nibabel module, img is an object that Nibabel creates which contains all the information from the file e.g., the header, the data and the affine. We can obtain all these using getter methods:


data = img.get_data()
affine = img.get_affine()
header = img.get_header()
voxel_size = header.get_zooms()[:3]


Here data is a Numpy array which contains the actual 4D image (a collection of 3D volumes). Using data.shape we can obtain the dimensions of the image. In this example, the dimensions are (81, 106, 76, 160). We can think of this 4D image as a sequence of 160 sequential 3D volumes, each containing (81, 106, 76) voxels. The variable affine provides a 4 × 4 matrix which contains the transformation matrix which maps 3D voxel image coordinates to world mm coordinates. This matrix can be useful when registering, saving or visualizing images. The voxel_size is a tuple with three values. In this example the voxel_size is (2, 2, 2), corresponding to a volume of 8 mm^3^.

Supposing that we want to extract and save from the 4D data only the first volume (usually this is the volume containing the non-diffusion-weighted data, also denoted as S0). We can do that very easily using the following code.


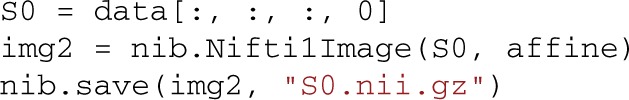


As we said previously data is a Numpy array. Numpy arrays provide simple ways to extract information from N-dimensional datasets, an operation known as slicing. For example, data[10:20, 15:25, 20:30, 30:50] returns a new sub-array with shape (10, 10, 10, 20) starting at (10, 15, 20). When a colon on its own (:) is used that means that all points in this dimension are used. The only delicate point here is that in order to save this new array we will need to update the affine by adding the sub-array's starting vector to the original offset vector. This is possible in the following way:


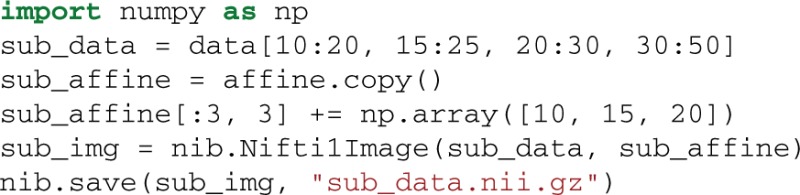


### 5.2. Background removal

**Figure 3:**
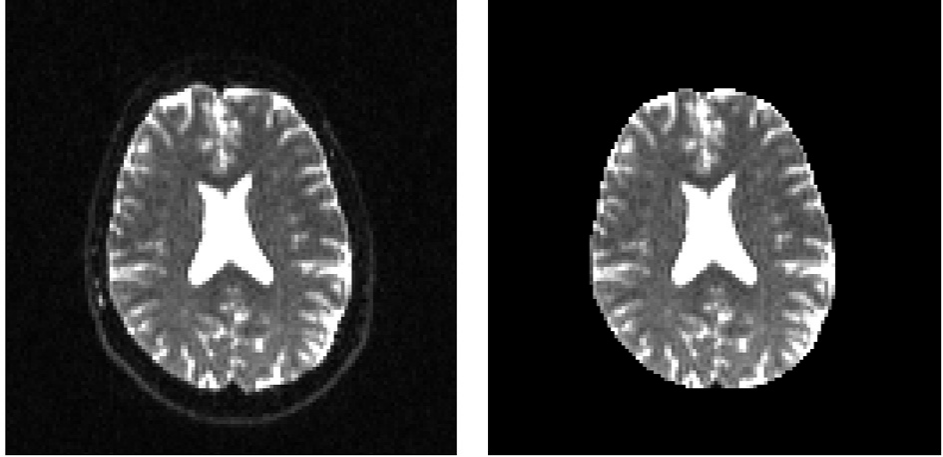
**Showing an axial slice in the center of S0 before (left) and after brain extraction (right) using median_otsu**.


In order to remove the background and keep only the parts that are in the brain we can use a function called median_otsu (see Figure 3).

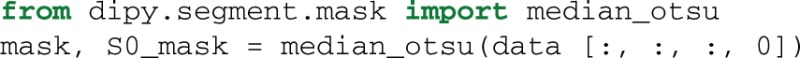


median_otsu uses first a median filter to smooth the S0 and then Otsu's method, an automated histogram method (Otsu, 1979) to separate the brain (foreground) from its background. It returns two arrays, the mask, a 3D array with 1s for the foreground and 0s for the background and the masked S0, S0_mask a 3D array with the actual S0 values in the foreground and 0s in the background.

### 5.3. Gradient table

The *b*-value *b* or *diffusion weighting* is a function of the amplitude, duration, temporal spacing and timing parameters of the specific paradigm. In the case of the classical Stejskal-Tanner pulsed gradient spin-echo (PGSE) sequence, the signal at the time of readout is given by:

(1) $$b=\gamma^{2}G^{2}\delta^{2}\left(\Delta -\delta /3\right)$$

γ is the gyromagnetic ratio, δ denotes the pulse width, *G* is the gradient amplitude and Δ is the center to center spacing. γ is a constant which depends on the type of nucleus and on the strength of the static magnetic field of the MRI machine, but we can control the *b*-value by changing the other three parameters. By changing the *b*-value along different gradient directions MR researchers can alter the quality and duration of the dMRI experiment. The gradient unit directions are often referred as *b*-vectors. We need to know the *b*-values and *b*-vectors for each 3D volume in order to analyze the diffusion data. These can usually be read from one or two different files. Here is an example:


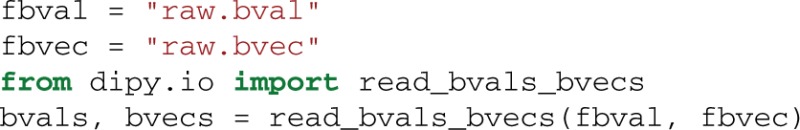


The *b*-value and *b*-vector parameters are stored in a utility class, created by the gradient_table function:


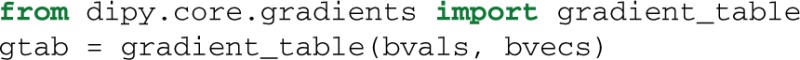


Here, gtab is an instance of a GradientTable object. This object checks the input values and provides the *b*-values and *b*-vectors in a form that Dipy knows how to use. For example, the shape of gtab.bvals and gtab.bvecs should always return *N* and *N* × 3, respectively, where N is equal to the size of the last dimension of data. Often, it is useful to know the number and position of *b*-values with value 0 (the b0 volumes); there is an utility method for this purpose—gtab.b0s_mask.

The GradientTable object can also store other acquisition parameters like time between volumes (Time-to-repeat, TR) and echo time (TE). These are Δ and δ, respectively in Equation (1). The GradientTable is therefore an abstract representation of the acquisition parameters.

## 6. Reconstruction

In diffusion MRI, the motion of water molecules is probed in a spatial- and direction-specific manner. That is, in every spatial location in the brain (typically sampled in voxels each covering a volume of approximately 2 × 2 × 2 mm^3^), the diffusion in several different directions is probed through the application of directional magnetic gradients. This motion due to diffusion takes place at a microscopic level, therefore, if we want to describe how the molecules diffuse we have to study this phenomenon from a statistical point of view. When a molecule is at position **x**_0_, we cannot read exactly where it will be after time *t*, we can only model a distribution of possible locations i.e., a probability displacement distribution. This is also known as the diffusion propagator *P*. This motion is described by the propagator *P*(**x**; **x**_0_, *t*) which defines the probability of being in **x** after a time *t*, starting at **x**_0_. Callaghan (1991) showed that the spin echo magnitude *S*(**q**, *t*) from a pulsed gradient spin echo (PGSE) experiment is directly related to the diffusion propagator by the following (inverse) Fourier relation

(2) $$S(q,t)=S_{0}\int P(r,t)e^{i2\pi q\cdot r}dr$$

where *S*_0_ is the signal in the absence of the applied magnetic diffusion gradient **g**, **r** is the relative spin displacement **x** − **x**_0_ at diffusion time *t* and **q** is the spin displacement wave vector. **q** is parallel with the applied magnetic gradient **g** which in turn are directly related with the *b*-vectors and *b*-values as discussed in section 5.3. In theory, if we apply the Fourier transform in Equation (2) we can get back to the probability displacement distribution (PDF) for every voxel. In practice, the first methods to calculate the PDF were q-space imaging (QSI) (Callaghan et al., 1988) and the most recent diffusion spectrum imaging (DSI) (Wedeen et al., 2005) (see section 6.2). DSI needs long acquisition time although recent techniques based on Compressed Sensing ideas (Menzel et al., 2011; Bilgic et al., 2012; Gramfort et al., 2013), and multi-slice imaging (Setsompop et al., 2012) have considerably accelerated the DSI acquisition. An alternative is to work on reconstructing only an angular projection of the 3D PDF. This reconstruction is often called the orientation distribution function (ODF). Another alternative is to make assumptions about the distribution of the PDF such as a Gaussian assumption, which leads to the popular diffusion tensor imaging (DTI) technique (Basser et al., 1994).

### 6.1. Diffusion tensor imaging

Often people assume that DTI and dMRI are synonyms. This is not correct. In DTI, there is a crucial assumption that the diffusion propagator is described only by a single 3D Gaussian distribution. From Equation (2), we can thus write

(3) $$P(r,t)=\frac{1}{\sqrt{4\pi t^{3}|D|}}\exp(-\frac{r^{T}D^{-1}r}{4t})$$

where **D** is known as the diffusion tensor. This tensor is a 3 × 3 positive definite symmetric matrix that can be completely described by a centered ellipsoid with three principal axes and associated eigenvalues λ_1_ ≥ λ_2_ ≥ λ_3_.

DTI was first proposed by Basser et al. (1994) and has since been very influential in demonstrating the utility of dMRI in characterizing properties of white matter tissue. The model relies on the assumption that diffusion is a Gaussian process, which can be captured by six parameters, describing the variance and covariance of the Gaussian diffusion along the three primary axes. From these parameters, several useful measures can be derived. The primary diffusion direction is the principal eigenvector of the tensor. It has eigenvalue λ_1_ and is the direction in which variance in the diffusion distribution is largest. In some places in the brain (e.g., in the corpus callosum, the large commissural fiber bundle connecting left and right hemispheres), this corresponds to the direction of the white matter fibers in the voxel and can be used for streamline tracking (Conturo et al., 1999; Mori et al., 1999; Basser et al., 2000). Other univariate measures can be estimated from the parameters of the tensor model to estimate the biophysical properties of the underlying tissue. The diffusivity along the primary direction (referred to as axial diffusivity, or AD) and along other directions (radial diffusivity, or RD), as well as the mean diffusivity (MD) are thought to index proportions of extra-cellular and intra-cellular water within the voxel. For example, MD is commonly used in the diagnosis of acute ischemic stroke, because these types of brain injury are characterized by cell-body swelling. The reduction in extracellular water fraction results in a decrease in MD in the affected regions (Maas and Mukherjee, 2005).

The fractional anisotropy (FA) is a normalized measure of the variation in diffusivity between different directions and was originally thought to index the organization of the tissue within the voxel (Basser and Pierpaoli, 1996). Later studies in animal models demonstrated that FA decreases when demyelination occurs (Song et al., 2002), because of increases in radial diffusivity. These studies showed that degree of myelination in the white matter is an important factor in limiting diffusion of water across cellular compartments. In addition, tissue density affects both the radial and the axial diffusivity, and loss of nerve fiber tissue also results in a decrease in FA (Beaulieu et al., 1996; Beaulieu, 2002). As a consequence of these studies, researchers now routinely refer to FA as a measure of *tissue integrity*. However, we strongly warn against this usage, because it is easy to show that although the density of the tissue and the density of the myelin wrapping the axons in the voxel may affect both MD and FA, changes in other factors, such as the distribution of directions of crossing of fiber populations through the voxel may also affect these measures (Basser and Pierpaoli, 1996; Jones et al., 2013; Wandell and Yeatman, 2013). Therefore, interpretation of group differences in FA, or longitudinal changes in FA over time should be carefully handled and compared to the results from other modeling techniques, that better account for the distribution of fiber orientations in the voxel (see below).

That said, the use of diffusion tensor-based univariate statistics is very popular among users of dMRI. Variance in a variety of behavioral (Ben-Shachar et al., 2007) and clinical (Thomason and Thompson, 2011) measures can be predicted based on these statistics, suggesting that they are reporting on meaningful variability in brain structure.

It is straightforward to use Dipy to fit the tensor model and compute univariate statistics from it. In the following example, we show how to fit the tensor model to data and how to compute univariate measures.

First, data, mask and gtab are created as we saw in section 5. Next, we can import the diffusion tensor model class and initialize a TensorModel class instance with the name ten_model:


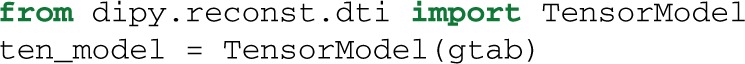


The code above sets up the analysis. Since the analysis of every voxel in the brain will rely on similar infra-structure, the TensorModel class instance only sets up the skeleton for the analysis. The same skeleton will be used for every voxel in the data here, but can also be used with new data in a similar fashion. To run the analysis, we pass the data to the fit method of the tensor_model. This returns a TensorFit class instance which relates to the specific data and mask:


ten_fit = ten_model.fit(data, mask)


The advantage of this separation of the model and the fit is that we can fit many different data with the same initialization parameters without duplicating code. Once a fit has been conducted, we can compute a variety of derived univariate measures, such as the fractional anisotropy (FA) (Basser and Pierpaoli, 1996):


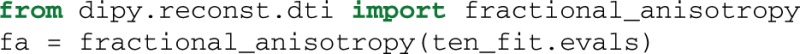


**Figure 4:**
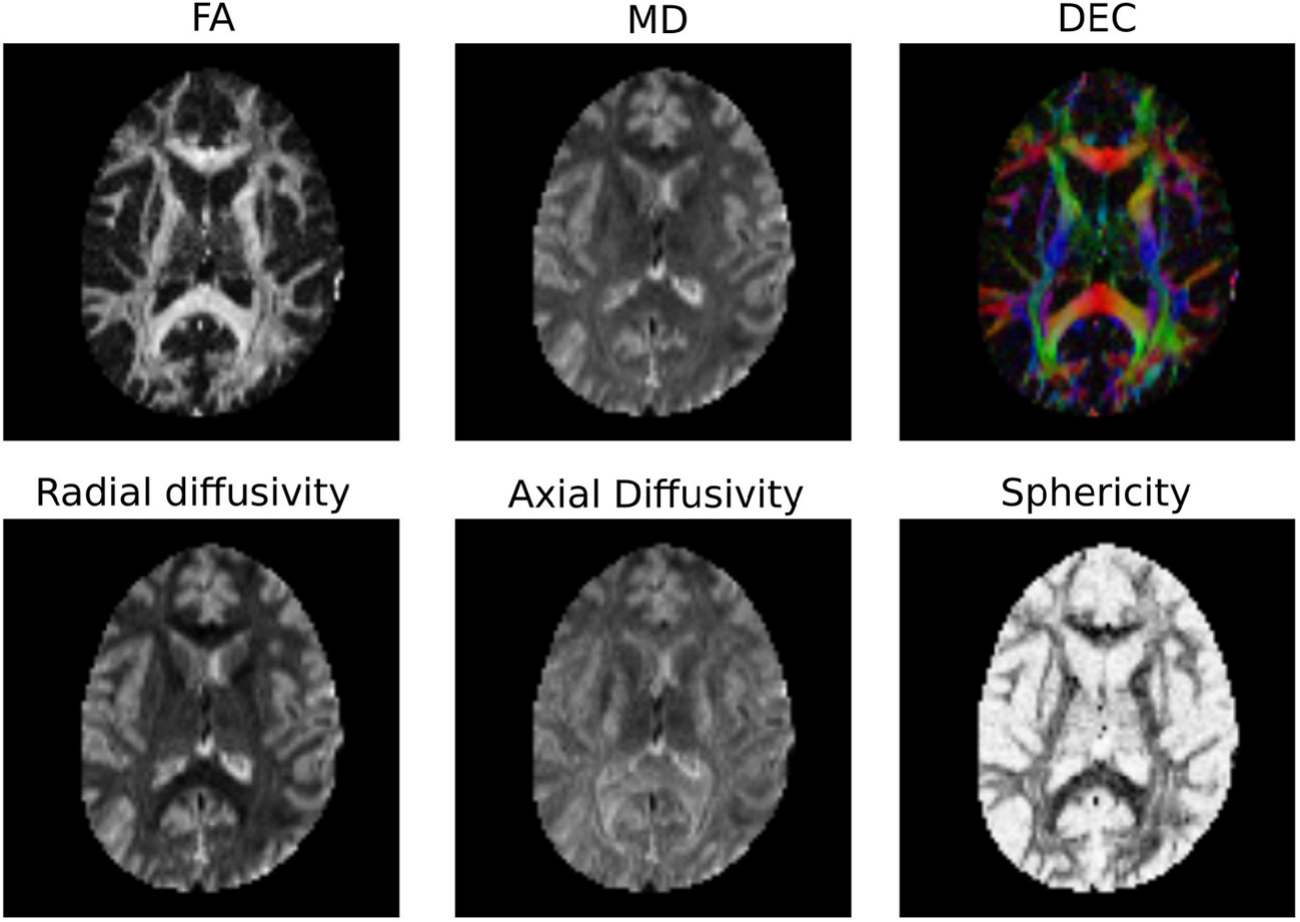
**Diffusion Tensor based scalar maps created with Dipy**.

It is common to represent the primary diffusion direction using a red-green-blue (RGB) representation and create a DEC (Directionally Encoded Color) map (Pierpaoli et al., 1996; Pajevic and Pierpaoli, 1999) which is also known as color FA (see Figure 4):


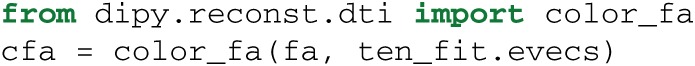


Finally, a couple more points about the implementation of DTI. The first is that there is still ongoing research on fitting methods for the tensor model (Koay et al., 2006). Several different methods have been implemented, including non-linear least-squares, ordinary least-squares, and weighted least-squares fitting (Chung et al., 2006) as well as Riemannian modeling-based techniques that assure that the estimated tensor is positive definite (Arsigny et al., 2006; Lenglet et al., 2006). In addition, there is ongoing research on methods to robustly fit the tensor model, in the face of noisy data (Chang et al., 2005, 2012). For this purpose, an implementation of a robust tensor fitting algorithm (RESTORE) is also available in Dipy.

Note also that Dipy implements many more univariate measures. Not only can users compute FA, MD, DEC, but also other statistics that have been proposed in the literature, such as the diffusion linearity, planarity and sphericity (Westin et al., 1997), as well as the tensor mode, tensor norm (Ennis and Kindlmann, 2006), radial and axial diffusivity. All these tensor-based metrics are available under the dipy.reconst.dti module. In Figure 4 a few of the many available measures are shown.

### 6.2. Diffusion spectrum imaging

For those who have acquired DSI data, i.e., data with multiple *b*-values and gradients that span a Cartesian keyhole grid (Tuch, 2002; Wedeen et al., 2005; Garyfallidis, 2012), Dipy currently provides three different models. The standard DiffusionSpectrumModel can be accessed using:


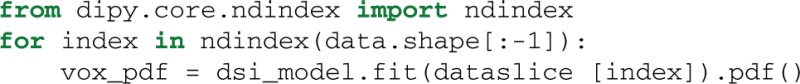


Wedeen et al. (2005) showed that the dMRI signal is positive for any type of spin motion without net flux (i.e., spin displacements due to thermal molecular agitation) or other random fluxes such as intravoxel incoherent motion. Under this assumption we can replace the complex signal *S* with its modulus |*S*| in Equation (2) and apply the Fourier transform:

(4) $$P(r)=S_{0}^{-1}\int |S(q)|\exp(-i2\pi q\cdot r)dq$$

In DSI this 3D-integral is approximated in a discrete way and the PDF *P* for every single voxel is returned as a 3D array. We can obtain *P* using:


dsi_fit = dsi_model.fit(data)
dsi_pdf = dsi_fit.pdf()


However, we recommend this method only when the dimensions of data are very small. This is because the dsi_pdf is a 6D array with 3 dimensions for the voxel positions and 3 for the **q** positions. For a moderate data set of 150 × 150 × 90 where every PDF is 35 × 35 × 35 we would need about 600 GB of RAM. As a solution to this problem Dipy provides a method which can facilitate traversing through each voxel and calculating each voxel independently:


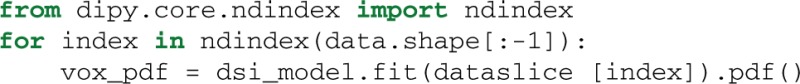


With this method we do not need to store all the PDFs at once. If it is necessary to save all the PDFs then our advice is to create a Numpy memory-map which will store the PDF for each voxel as a binary file on disk while still appearing as an array in memory. Memory-mapped files are used for accessing small segments of large files on disk, without reading the entire file into memory.

Recently, an alternative method for DSI was proposed by Canales-Rodríguez et al. (2010) using a deconvolution technique based on a Lucy–Richardson (LR) algorithm of the 3D PDF. The deconvolution technique accounts for the truncation of q-space by standard DSI and can thus achieve a higher angular resolution for resolving crossing fibers. This can be used in exactly the same way as the standard DSI method:


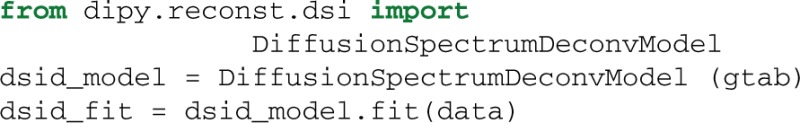


**Figure 5:**
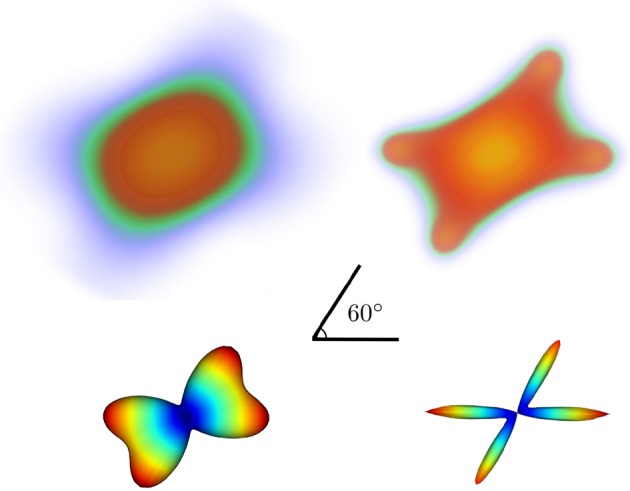
**Volumetric rendering of the 3D diffusion propagator of a 60° crossing with standard DSI (top-left) and DSI with deconvolution (top-right) with their corresponding ODFs (bottom)**.

In Figure 5 we show noiseless volumetric renderings of the PDFs of a simulation of a 60° crossing with the two different methods. It is evident that the LR deconvolution reconstruction better represents the underlying structure.

Since we are mainly interested in the angular structure of the underlying tissue, we further simplify the data by taking the weighted radial summation of *P*(**r**)

(5) $$\psi_{\text{DSI}}(\hat{u})=\int_{0}^{\infty }P(r\hat{u})r^{2}dr$$

This defines the orientation density function (ODF) for DSI which measures the quantity of diffusion in the direction of the unit vector **û** where **r** = *r***û**. ψ_*DSI*_ is a function on a sphere. Therefore, in order to calculate it or even visualize it we will need a set of spherical coordinates. Here is how we can obtain this ODF:


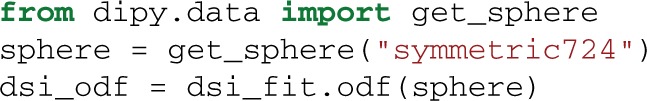


where sphere is an object holding the vertices and faces of the sphere. Here we used a symmetric sphere with 724 vertices. The dsi_odf is an array of the 724 ODF values corresponding to the vertices of the sphere. Note at this point that in order to find the ODF we have to first create the diffusion propagator (PDF) by applying the Fourier transform on the lattice (see Figure 5). Nonetheless, Dipy, for reasons explained before does not store the PDFs as it computes the ODF. Yeh et al. (2010) proposed a direct way (GQI) to calculate a slightly different ODF using the Cosine transform. GQI is available from dipy.reconst.gqi.GeneralizedQSamplingModel. The advantage of GQI is that it is much faster to calculate than DSI or DSI with deconvolution.

### 6.3. Q-ball imaging

To reduce the acquisition requirements of DSI, several techniques have been proposed to compute the ODF from Equation (5) using only a single *b*-value dMRI acquisition, often called *single-shell* high angular resolution diffusion imaging (HARDI) (Tuch et al., 2002; Descoteaux et al., 2011). Spherical harmonics (SH) are mathematical functions that provide a complete orthonormal basis for functions on the sphere. In practice, these can be used to approximate any spherical function (such as the ODF) up to a highest frequency (SH order). Due to their practical mathematical properties, there have been several proposals using spherical harmonics to describe the apparent diffusion coefficient (ADC) computed from HARDI, starting from the work of Frank (2001, 2002) and Alexander et al. (2002) and Tuch et al. (2002). Then, Tuch showed that the Funk Radon Transform (FRT), used in a method he called *q-ball imaging* (QBI), reconstructs a smoothed version the ODF directly from a *single-shell* dMRI acquisition. This q-ball ODF ψ_*QBI*_ can be obtained analytically from a SH estimation of the diffusion signal (Anderson, 2005; Hess et al., 2006; Descoteaux et al., 2007):

(6) $$\psi_{\text{QBI}}(\theta ,\phi )=\sum_{j=1}^{R}2\pi \frac{c_{j}}{S_{0}}P_{l(j)}(0)Y_{j}(\theta ,\phi )$$

where *P*_*l*(*j*)_ is the Legendre polynomial of order *l* corresponding to coefficient *j* and the coefficients have been normalized by the S0 (non-diffusion weighted) image. Hence, the q-ball ODF is a linear transformation of the SH coefficient, *c*_*j*_. This technique is called analytical QBI (aQBI), in contrast to the original QBI solution, which performs the FRT numerically. It is important to note that these solutions are based on Equation (5), without the *r*^2^ term. So these ODFs are not properly normalized. Hence, Dipy also implements the Constant Solid Angle analytical solution more recently proposed by Aganj et al. (2010) and Tristan-Vega et al. (2009).

The SH of order *l* and phase *m*, *Y*^*m*^_*l*_(θ, ϕ), arises from the angular solution to Laplace's equation in spherical coordinates and they form an orthonormal basis for complex functions defined on the unit sphere. However, in single-shell acquisitions, S is real and symmetric. Hence, it is common to define a real and symmetric modified orthonormal SH basis, *Y*_*j*_, using only even order terms and real/imaginary parts of *Y*^*m*^_*l*_(θ, ϕ). Therefore, the measured signal S is estimated with a truncated SH series of order *l*_max_, which has *R* = (*l*_max_ + 1)(*l*_max_ + 2)/2 terms. For example, for *l*_max_ = 4, 6, 8, and 16 SH series have *R* = 15, 28, 45, and 153 coefficients, respectively.

In Dipy we have implemented three different Q-ball methods in the module dipy.reconst.shm: (a) Descoteaux et al. (2007), (b) Aganj et al. (2010), and (c) Tristan-Vega et al. (2009). For example (a) can be used in the following way:


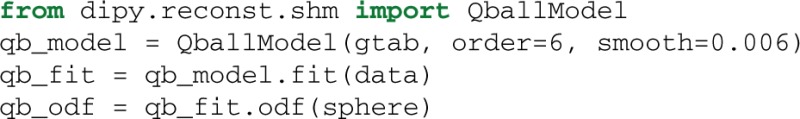


Important points to note are that SH order is set at 6 and the regularization parameter at 0.006, following the optimization recommendations of Descoteaux et al. (2006). Furthermore, because the analytical ODF method produces smoother ODFs it is useful for visualization purposes to use *min-max* normalization.


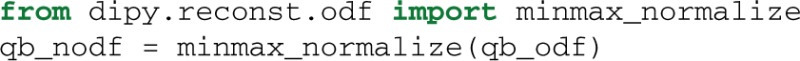


The SH coefficients are accessible using the attribute qb_fit.shm_coeff. Methods (b) and (c) can be used in a very similar way. For example, for the Constant Solid Angle (CSA) (Aganj et al., 2010) method (b) we only need to remove the normalization function and reduce the SH order as the CSA method becomes considerably noisier in higher orders:


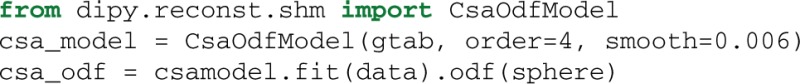


As a side note: the term Constant Solid Angle derives from the fact that this method calculates the ODF taking account of radial distance as we see in Equation (5).

### 6.4. Constrained spherical deconvolution

QBI-based techniques reconstruct the *diffusion ODF* (dODF). To improve the angular resolution of the reconstruction, spherical deconvolution (SD) techniques have been introduced and reconstruct what is called the *fiber ODF* (fODF). SD was first introduced by Tournier et al. (2004). With this method, the signal measured on single spherical shell acquisitions can be expressed as the convolution over spherical coordinates of the response function with the fODF. The response function describes the signal intensity that would be measured as a function of orientation for a single fiber aligned along the *z*-axis. In the spherical harmonics (SH) framework, the convolution operation is performed as follows. For each harmonic order *l*, the SH coefficients of the signal profile *S*(θ, ϕ) and the fODF *F*(θ, ϕ) are written as vectors **s**_*l*_ and **f**_*l*_ of length 2*l* + 1, whereas the rotational harmonic coefficients of the convolution kernel *R* (the response function) are written as a matrix **R**_*l*_ of size (2*l* + 1) × (2*l* + 1). The convolution operation then simply consists of one matrix multiplication per harmonic order *l*: **s**_*l*_ = **R** · **f**_*l*_. The spherical deconvolution operation can be performed by simple matrix inversion. However, the spherical deconvolution problem is ill-posed and thus severely affected by noise (Tournier et al., 2004).

Constrained super-resolved spherical deconvolution (CSD) Tournier et al. (2007) gives a robust solution to this problem by applying two major constraints on the fitting of the fODF. The first is that it applies a non-negativity constraint: fODF values that are smaller than 0 are non-physical and are precluded. The other is that CSD assumes that only a few of the fODF values will be large. Applying these two constraints allows fitting the SH basis up to very high orders, in essence fitting more parameters than the data allows. This super-resolved method can be accessed in Dipy using:


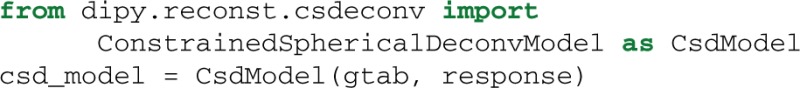


The main choice to consider is the estimation of the single fiber response function. We assume that *R* is derived from a prolate tensor, where the single-tensor model is accurate. The eigenvalues of this tensor are estimated from the voxels with FA > 0.7. The input parameter response is a tuple with two parameters: (a) the eigen-values of the tensor and (b) the estimated average S0 signal for those voxels. The response function is usually estimated from the corpus callosum areas. For further information on how to initialize the CSD please read our examples at dipy.org.

Dipy also implements a second constrained spherical deconvolution method: the Spherical Deconvolution Transform (SDT) (Descoteaux et al., 2009), which is a sharpening operation that can transform the smooth diffusion ODF into a sharper fiber ODF. The method is inspired by CSD (Tournier et al., 2007) with the main difference that the CSD is applied directly to the initial signal and the SDT directly to the ODF (Descoteaux, 2008; Descoteaux et al., 2009).

For the derivation and explanation of the formula see Descoteaux et al. (2009). You can use the SDT in the following way:


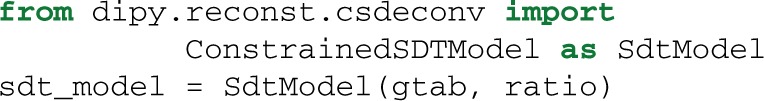


Here the response function is provided as a scalar parameter ratio, which is the ratio of the smallest eigenvalue to the largest eigenvalue. Both spherical deconvolution methods perform similarly as shown in Descoteaux et al. (2009) and Garyfallidis et al. (2013).

**Figure 6:**
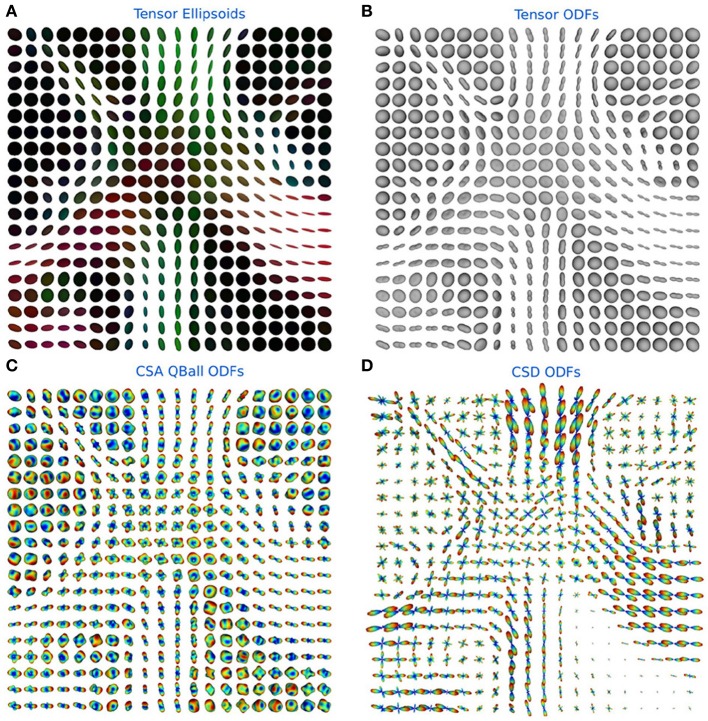
**(A)** Tensor ellipsoids color-coded with a DEC map, **(B)** tensor ODFs, **(C)** constant solid angle ODFs, and **(D)** constrained spherical deconvolution ODFs in a region in the centrum semiovale showing crossings between the corpus callosum, corticospinal tract and the superior longitudinal fasciculus.

In Figure 6 the ODFs of the TensorModel, CsaOdfModel and CsdModel of a region in the centrum semiovale show crossings between the corpus callosum, corticospinal tract and the superior longitudinal fasciculus.

### 6.5. Peaks from models

In the previous sections we showed that the reconstruction models have a uniform API and can be called in similar ways. For example, they all have an odf() method. This design gives us the opportunity to create utility functions where the model is one of the parameters. peaks_from_model is a multipurpose function which can be used to (a) find the maxima (peaks) of the ODFs, (b) find the directions of the maxima in the ODFs (which can be useful for tracking), (c) discretize those directions on the unit sphere for efficiency, (d) compress the ODFs as spherical harmonics to reduce memory usage, and (e) calculate many metrics simultaneously—e.g., generalized fractional anisotropy (GFA) (Tuch, 2004)—without the need to create all ODFs at once. peaks_from_model can be called as:


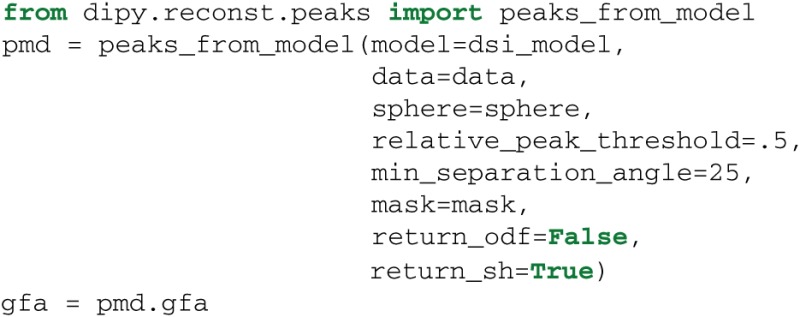


The model parameter can be set to any of the models discussed in the previous sections e.g., dsi_model. The relative_peak_threshold parameter specifies that only peaks greater than relative_peak_threshold∗*m* should be returned, where *m* is the value of the largest peak. min_separation_angle sets the threshold for the minimum angular distance in degrees between two peaks. If the peaks are closer than this threshold only the larger of the two is returned. These two parameters help to get robust fiber directions when the ODFs are noisy. peaks_from_model returns a PeaksAndMetrics object which holds all the different output arrays, peak_values, peak_indices, gfa, qa (Yeh et al., 2010), odf and shm_coeff.

If the parameter return_sh is set to True then the ODFs will be represented by their SH expansion. This reduces memory usage, as the SH coefficients need much less memory than the ODF represented on the sphere. If we want to calculate the ODF back from the SH coefficients we can use the function sh_to_sf:


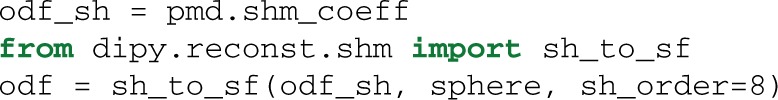


## 7. Fiber tracking

One of the tantalizing prospects of dMRI is that combining information about local microstructure across different voxels may provide information about large-scale organization of the brain. In particular, researchers have been using various algorithms in attempts to track along the presumed fiber populations to make inferences about axonal connections between different parts of the gray matter (see Figure 1). The connection matrix that results from an exhaustive connectivity analysis of all parts of the cerebral cortex is sometimes referred to as a *connectome* (Sporns et al., 2005) and understanding the structure and function of the connectome is a central goal of contemporary neuroscience. This premise has led to major investment in data-collection projects aimed at characterizing the connectome, based on dMRI, as well as functional MRI (fMRI) measurements (Van Essen et al., 2013).

Tracking algorithms used to infer these connections divide into two major classes. The first class is deterministic. Deterministic streamlines follow a predictable path through the data, by selecting at each point a single diffusion direction to follow. There may be several estimated directions at each point (such as a voxel center), but deterministic selects one of these estimated directions on some criterion such as closeness of match to the previous direction of the streamline. Some of the early deterministic algorithms used the primary diffusion direction of the diffusion tensor model as an indication of the direction of the major fiber population in every voxel and track along the streamlines that are implied by these principal diffusion directions (Conturo et al., 1999; Mori et al., 1999; Basser et al., 2000). However, it is now widely recognized that in regions of crossing fibers, the primary diffusion direction may not coincide with the direction of any of the local fiber populations. More modern algorithms (see below) take this into account.

The second major class of tractography algorithms is probabilistic. These methods consider the local information in each voxel to represent a distribution of possible directions in that location. In these algorithms, a trajectory of fibers in every location is randomly sampled from the local distribution. Any given streamline in a probabilistic tractography is therefore one random sample of many possible streamlines. The algorithm may therefore give different sets of streamlines on different realizations.

Dipy implements both deterministic and probabilistic tracking algorithms, described in detail below.

### 7.1. Deterministic

Euler Delta Crossings (Garyfallidis, 2012), or *EuDX* for short, was the first tracking method to be implemented in Dipy. We created an algorithm that has many similarities with the classical deterministic methods (Conturo et al., 1999; Mori et al., 1999; Basser et al., 2000) and with more recent ones such as those described in Descoteaux et al. (2009) and Yeh et al. (2010). Our focus was on creating a simple deterministic algorithm which can be used with very different families of reconstruction models, work well in crossing areas and be efficient so that it can be used to quickly inspect the reconstruction results. EuDX is usually applied in native space image coordinates and it assumes that voxels are of equal size in all three image axes (isotropic voxel size). If the raw data does not have isotropic voxel size then a reslicing preprocessing step is required to make the data isotropic.

In order to create streamlines, we initially need to provide one or more seed points **s**. The seed points are the points from which the streamlines will start growing. These can be chosen randomly or they can be specified explicitly. However, the seed points need to be constrained by the volume's dimensions. Every seed point **p**_0_ becomes the starting point for the track propagation. For the integration we solve for **p**_*t*_ = **p**_0_ + ∫^*t*^_0_ **v**(**p**(**s**))*d***s** and we perform the integration numerically using Euler's method

(7) $$p_{n+1}=p_{n}+v(p_{n})\Delta s$$

where Δ*s* is the propagation step size (which should be no greater than the voxel size), and **v** is the propagation direction. EuDX uses trilinear interpolation for the calculation of the next direction, integrating directional information from the surrounding voxels.

The first parameter of EuDX can be an array of dimensions *X* × *Y* × *Z* like FA or *X* × *Y* × *Z* × *W* like the ODF, or quantitative anisotropy (QA) (Yeh et al., 2010). These arrays can be used for stopping the propagation if the value in the current voxel is lower than a_low. For efficiency, the peak directions are discretized on a unit sphere. For this purpose, the second parameter is another array of dimensions *X* × *Y* × *Z* or *X* × *Y* × *Z* × *W* but this time these are the indices of the directions approximated on the sphere. Here we give an example where we have used the peak_indices from CSD using peaks_from_models and the tensor FA for stopping criteria (with threshold 0.1):


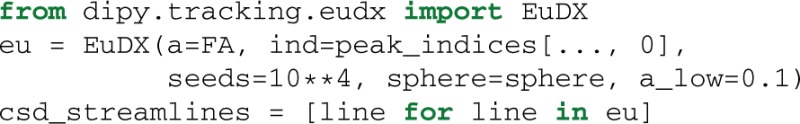


**Figure 7:**
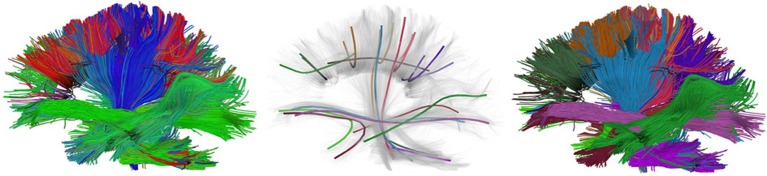
**Left:** An example of EuDX streamline tracking applied on a real human brain dataset and color-coded with a standard orientation colormap. **Middle:** Showing the QuickBundles centroids with random colors. **Right:** Showing the clusters color-coded with their corresponding centroid color.

The input parameter seeds can be given as an integer, this will generate random seeds in the entire volume, or it can be given as a *N* × 3 array of seed points. The latter explicit specification of seed points has the advantage that it allows us to seed from specific ROIs or from the gray matter—white matter boundary which has been shown to generate more robust tracking results (Côté et al., 2013). The instance of EuDX returns an iterator. In every call of the iterator a new streamline is returned. This technique allows one to generate and save streamlines directly to disk without loading all streamlines in memory. In Figure 7 a few thousand human brain streamlines are shown, approximating the brain's white matter connections, generated from EuDX. In contrast, in Figure 1 only a specific anatomical bundle is approximated rather than the full brain.

### 7.2. Probabilistic

Probabilistic fiber tracking describes a class of tractography algorithms that estimate multiple possible pathways though each point by taking into account the uncertainty in fiber direction at those points. The uncertainty in fiber direction can be estimated as an fODF, this distribution can be used along with a Monte Carlo sampling to generate streamlines (Morris et al., 2008). Streamlines are generated using a Markov process which is similar to deterministic fiber tracking. Streamlines begin at a seed point and continue along a propagation direction which is randomly chosen from the fODF. The propagation direction is continually adjusted by sampling from the fODF at each new location along the path. This continues until some stopping criteria are met. Notice that this framework is equivalent to deterministic fiber tracking if the fODF at each point is a delta function of one fiber direction.

Dipy has implemented two interfaces for probabilistic Markov fiber tracking. The first method allows the user to provide the distribution evaluated on a discrete set of possible tracking directions. For example the fiber orientation distribution function (fODF) obtained by fitting the CSD (constrained spherical deconvolution) model to diffusion data can be used as an estimate of the PDF, because it represents a fiber distribution associated with the measured diffusion signal (Jeurissen et al., 2011). The user must also provide a set of seeds to track from, and set the stopping criteria. Currently a white matter mask and maximum turning angle are used as stopping criteria.

The second interface for probabilistic tracking is meant to accommodate tracking methods where the fODF cannot be easily computed. It is often much easier and less computationally expensive to sample from a PDF than it is to evaluate the PDF. For example, residual bootstrap fiber tracking methods fall into this category (Berman et al., 2008). In this case we combine a sampling method with the diffusion data and a diffusion Model, and pass this to the Markov tracking framework. Streamlines can now be generated as before without explicitly evaluating the orientation distribution at each point in the diffusion data. This framework is designed to be flexible and easily customizable so that a wide variety of tracking methods can be expressed.

## 8. Fiber analysis and post-processing

In the following sections we describe some of the tools available for processing streamlines after these have been created.

### 8.1. Streamline clustering

Depending on the initial number of seeds and other tracking parameters, fiber tracking algorithms can generate a great number of densely packed streamlines (often more than one million) which are difficult to interact with and interpret. As a solution to this problem Dipy implements a recent efficient clustering algorithm for streamlines called QuickBundles (QB) (Garyfallidis, 2012; Garyfallidis et al., 2012). QB can be used to simplify large datasets in a couple of minutes. When using QB we need first to instantiate the QuickBundles object with three parameters. The first parameter is the initial set of streamlines to be clustered, the second is the distance threshold that will determine the number of clusters and their sizes, and the third is the level of detail for each streamline. For example, in the code snippet below we use pts=18 which means that before QB starts the clustering procedure the streamlines will be downsampled so that each one has the same number of points (here 18) and equal length segments. This preprocessing step is a prerequisite for QB.


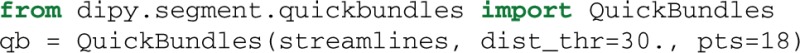


After we have created the instance of the object, attributes like qb.centroids provide the clusters' centroids and methods like qb.label2tracks() can return the streamlines which belong to a specific cluster. Figure 7 shows an example of QuickBundles applied on the human brain dataset described in Fortin et al. (2012), using the same parameters shown in the code listing above.

### 8.2. ROIs and streamline intersections

It is often useful in dMRI to filter, group or count streamlines based on their interactions with one or several ROIs (Côté et al., 2013). There are a few functions in dipy.tracking.utils to make these kinds of operations easier. The first of these functions, density_map, counts the number of streamlines that pass though each voxel and returns the result as an image. In all the following examples streamlines is a sequence of streamlines and affine is a mapping from voxel coordinates to world coordinates. In all cases affine can be omitted if the streamlines are defined in voxel coordinate space.


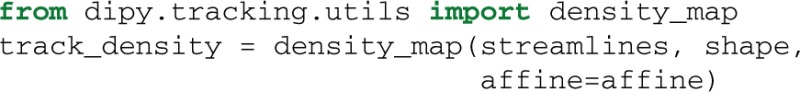


In this example shape is the shape of the 3D image to be returned, and track_density is a 3D volume where the intensity of each voxel is the number of streamlines that pass though that voxel. Note that each streamline is counted once per voxel even if multiple points in the streamline lie in that voxel.

Another useful function is target. This function filters a sequence of streamlines and keeps only those that pass through an ROI.


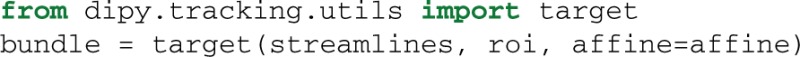


Here roi is a binary array, and bundle is generator of streamlines. Only the streamlines that pass though at least one of the voxels in roi that have value 1, will be in bundle.

streamline_mapping is a function related to target. This function produces a mapping from voxel indices to streamlines, much like targeting on every individual voxel, and returns a dictionary of those mappings.


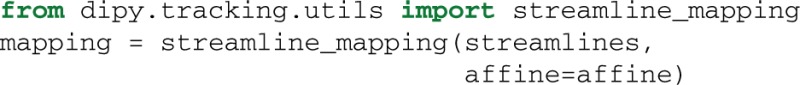


Here mapping is a dictionary where the keys are voxel indices and the value associated with each key is a list of all the streamlines that pass though that voxel. For example to get all the streamlines that pass though voxel (i, j, k), we would look up mapping[i, j, k].

The last function we want to mention in this section is connectivity_matrix. This function groups and counts streamlines based on their endpoints and a label volume which we call labels. This label volume should be an image where the intensities of the image map to anatomical structures. For example:


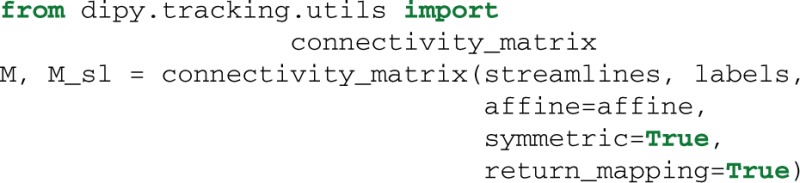


Here because we have made the matrix symmetric, M[i, j] = M[j, i] is the number of streamlines that connect region i to j or j to i. Similarly because we have set return_mapping=True, we can get a list of all those streamlines by looking up M_sl[i, j].

While it is common to apply these kinds of operations in native voxel space of the diffusion MRI images used to create the streamlines, this is not required. It is often useful to interact with streamlines in the voxel space of a high resolution structural image of the same patient. This is possible as long as one can compute a linear transformation (affine) between the voxel coordinates and the streamline point coordinates.

**Figure 8:**
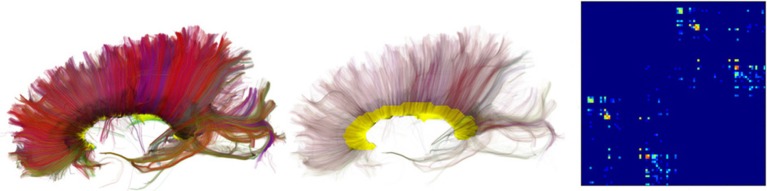
**Left:** Streamlines intersecting a mask in the corpus callosum (CC). **Middle:** Showing the streamlines with semi-transparency to make it easy to see the mask (yellow color). **Right:** Connectivity matrix of the CC streamlines that connect 89 cortical regions. A detailed tutorial which explains how it is possible to create similar connectivity matrices with your data is available at Dipy's website.

An example of using target and connectivity_matrix is shown in Figure 8. In the left panel we can see the streamlines found to intersect with the yellow mask in the corpus callosum (CC) using target. Then we used these streamlines to investigate which areas of the cortex are connected using a modified aparc+aseg.mgz label map created by FreeSurfer (Fischl, 2012) of 89 regions. For a complete example of how you can create your own connectivity matrices we recommend reading the online tutorial on the topic from Dipy's website^13^.

### 8.3. Streamline metrics and statistics

In Dipy, we have implemented several metrics for streamlines. For example, perhaps someone may want to calculate the average length and standard deviation of the streamlines generated after the fiber tracking procedure. This can be achieved very easily using the length function which takes as input a single streamline. We can then iterate through all the streamlines in the following way:


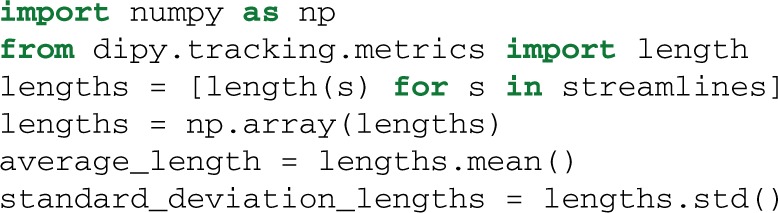


Many other metrics can be found in the metrics sub-module e.g., spline for spline interpolation, centre_of_mass, mean_curvature, mean_orientation and the frenet_serret framework for curvature and torsion calculations along a streamline.

### 8.4. Visualization

Figures 1, 4–7, are generated by our own visualization tools which can be used for most parts of the diffusion analysis pipeline. We have developed a minimal and lightweight module called fvtk which is based on the Visualization Toolkit (VTK) (Schroeder et al., 2001). The main idea is that a window can have one or more renderers. A renderer can have none, one or more actors. Examples of actors are a sphere, line, point or a complete set of streamlines. You can add actors in a renderer and in that way you can visualize the aforementioned objects e.g., sphere, line etc. The windows can be created by either the show function which creates a visible window or the record function which creates a temporary window only for the purpose of using it to render the objects and save the frames on disk. The renderer holds all the actors i.e., the visible objects. Here is a simple example where we visualize some streamlines with different colors:


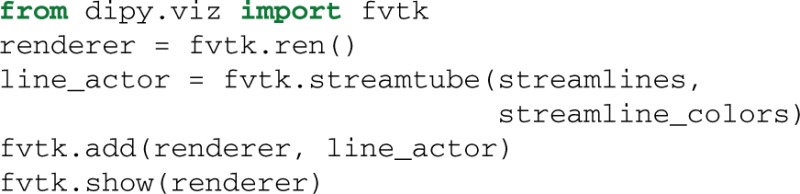


The function streamtube was also the one we used to create Figures 1, 7. The most commonly used visualization functions are given in Table 1.

**Table 1:** **List of visualization functions**.

**Name**	**Usage**
ren	Create renderer
add	Add actor to renderer
rm	Remove actor from renderer
rm_all	Remove all actors from renderer
show	Create window and show renderer
record	Save frame or frames
line	Creates an actor of one or more streamlines
streamtube	Same as line but with streamtubes
point	Creates actor of points as small spheres
tensor	Actor for tensor ellipsoids visualization
sphere_funcs	Actor for ODF visualization
volume	3D volume rendering with raycasting
slicer	Actor for showing volumetric slices

## 9. Discussion and conclusion

We have outlined the structure and functionality of the Dipy library. Dipy provides a number of simple-to-use methods for the analysis of diffusion MRI data using the Python language. We demonstrated examples of pre-processing, reconstruction, tracking and post-processing, showing that Dipy, although a relatively new project, can fill many of the steps needed to do a complete dMRI analysis. We have illustrated the scope of the methods that have been implemented to date, and have demonstrated these capabilities of Dipy with sample scripts and visualizations.

In the future, we are hoping to introduce new methods which are not currently implemented. Here are some hints about the areas that we are currently working on. For the pre-processing steps, we are currently implementing denoising and eddy-current correction of the raw dMRI data. For group analysis, we are creating novel warping algorithms. We have also started looking into tissue characterization in microstructure (Assaf et al., 2008). For the reconstruction, we have already a first implementation of SHORE (Özarslan et al., 2013) which we are currently enhancing with more features. For tracking, we are looking into building a more general and adaptive interface. For connectivity analysis, we are working with the Connectome Mapper (CM) (Daducci et al., 2012) developers to further integrate Dipy into the CM pipeline.

As the size of the datasets in laboratories and hospitals around the world keeps increasing it is very important to use parallel processing to reduce the duration of analysis. For this purpose we have already implemented a parallel execution for the reconstruction step which is usually a bottleneck of the overall dMRI analysis. This is enabled through peaks_from_model by setting the parameter parallel=True. Nonetheless, we are working on parallelizing other bottlenecks of the pipeline either by using multi-processing or OpenMP through Cython. Of course, for those who are interested in computing every subject in parallel e.g., in a cluster or in a multi-core computer this is very easy to do with Python and Dipy and there are many tools which can facilitate this e.g., IPython, Nipype, and others.

Dipy is free and open source software and it is part of a larger community found at nipy.org. This is a growing team of scientists and developers focusing on sharing code for different modalities of brain imaging. In common with the other Nipy projects, Dipy is being developed under the umbrella of a single GitHub organization^14^ and the central Dipy GitHub repository is managed under this organization^15^. The community provides support for the use and development of these software tools through the project's mailing list^16^.

**Figure 9:**
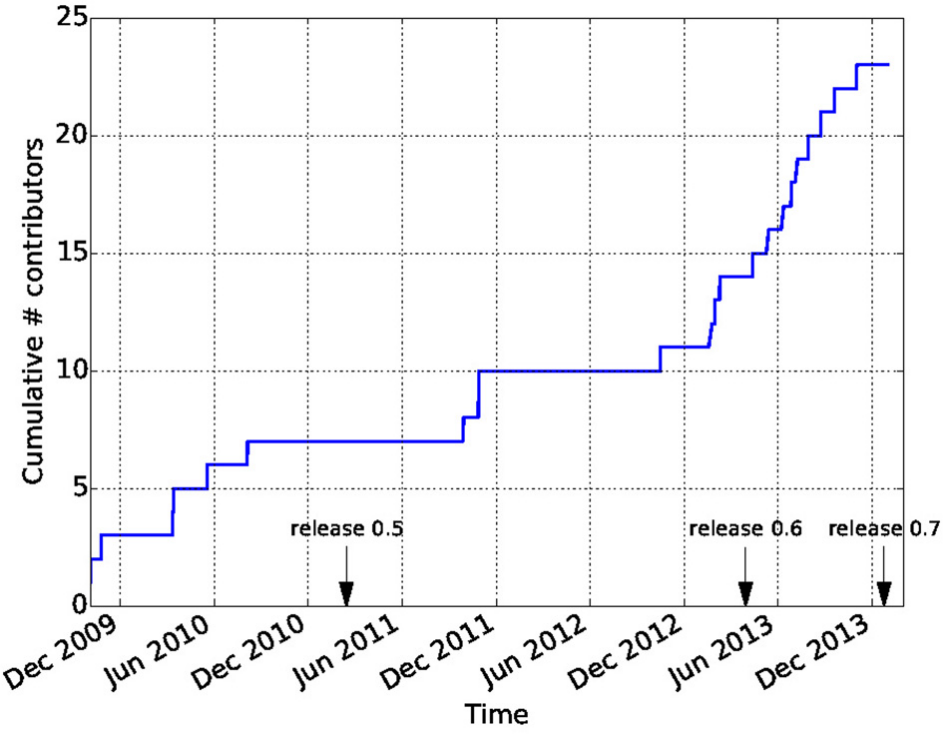
**Participation in the Dipy GitHub repository.** The cumulative number of unique contributors has been extracted using the git log command and tallied. Many new contributors have joined the project after the 0.6 release.

Github is widely recognized as a factor in lowering the barriers on participation in open-source software development. In combination with the principles of the Nipy community, this has led to a vibrant and diverse developer community. In contrast to many other software projects in neuroimaging, Dipy is not based on the work of one lab, or one institution. Though many of the contributions are made by a few core contributors, there are many contributors to the code-base and the number of contributors has been growing dramatically after the release of Dipy 0.6 which took place on April 2013 (see Figure 9). Dipy contributors come from at least seven different academic institutions in five countries (Canada, UK, USA, Italy, and South Africa).

In conclusion, we hope this paper inspires you to share our excitement in developing Dipy and encourages you to participate in the project. We strongly hope that more scientists will join Dipy by using the software and giving us feedback so that we can make it better. Stronger still is our hope that many will chose to share their code implementing new methods, and join the developer team. We are sure that a wide and open participation is absolutely necessary in order to solve the hard problems of brain mapping.

### Conflict of interest statement

The authors declare that the research was conducted in the absence of any commercial or financial relationships that could be construed as a potential conflict of interest.

## Footnotes

^1^http://nipy.org

^2^http://numpy.org

^3^http://scipy.org

^4^http://cython.org

^5^http://nipy.org/nibabel

^6^http://matplotlib.org

^7^http://vtk.org

^8^http://nose.readthedocs.org/

^9^http://sphinx-doc.org/

^10^http://ipython.org

^11^http://github.com/nipy/dipy

^12^http://buildbot.net

^13^http://dipy.org/examples_index.html

^14^http://github.com/nipy

^15^http://github.com/nipy/dipy

^16^http://dipy.org/subscribe.html

## References

[B1] Aganj I. Lenglet C. Sapiro G. Yacoub E. Ugurbil K. Harel N. (2010). Reconstruction of the orientation distribution function in single- and multiple-shell q-ball imaging within constant solid angle. Magn. Reson. Med. 64, 554–556 10.1002/mrm.22365 20535807 PMC2911516

[B2] Alexander D. Barker G. Arridge S. (2002). Detection and modeling of non-gaussian apparent diffusion coefficient profiles in human brain data. Magn. Reson. Med. 48, 331–340 10.1002/mrm.10209 12210942

[B3] Anderson A. (2005). Measurements of fiber orientation distributions using high angular resolution diffusion imaging. Magnet. Reson. Med. 54, 1194–1206 10.1002/mrm.20667 16161109

[B4] Arsigny V. Fillard P. Pennec X. Ayache N. (2006). Log-euclidean metrics for fast and simple calculus on diffusion tensors. Magn. Reson. Med. 56, 411–421 10.1002/mrm.20965 16788917

[B5] Assaf Y. Blumenfeld-Katzir T. Yovel Y. Basser P. J. (2008). Axcaliber: a method for measuring axon diameter distribution from diffusion mri. Magn. Reson. Med. 59, 1347–1354 10.1002/mrm.21577 18506799 PMC4667732

[B6] Basser P. Mattiello J. LeBihan D. (1994). MR diffusion tensor spectroscopy and imaging. Biophys. J. 66, 259–267 10.1016/S0006-3495(94)80775-1 8130344 PMC1275686

[B7] Basser P. J. Pajevic S. Pierpaoli C. Duda J. Aldroubi A. (2000). *In vivo* fiber tractography using DT-MRI data. Magn. Reson. Med. 44, 625–632 10.1002/1522-2594(200010)44:4<625::AID-MRM17>3.0.CO;2-O 11025519

[B8] Basser P. J. Pierpaoli C. (1996). Microstructural and physiological features of tissues elucidated by quantitative-diffusion-tensor MRI. J. Magn. Reson. 213, 560–570 10.1016/j.jmr.2011.09.022 22152371

[B9] Beaulieu C. (2002). The basis of anisotropic water diffusion in the nervous system - a technical review. NMR Biomed. 15, 435–455 10.1002/nbm.782 12489094

[B10] Beaulieu C. Does M. D. Snyder R. E. Allen P. S. (1996). Changes in water diffusion due to Wallerian degeneration in peripheral nerve. Magn. Reson. Med. 36, 627–631 10.1002/mrm.1910360419 8892217

[B11] Behrens T. Johansen-Berg H. (eds.). (2009). Diffusion MRI. Elsevier

[B12] Ben-Shachar M. Dougherty R. F. Wandell B. A. (2007). White matter pathways in reading. Curr. Opin. Neurobiol. 17, 258–270 10.1016/j.conb.2007.03.006 17379499

[B13] Berman J. Chung S. Mukherjee P. Hess C. Han E. Henry R. (2008). Probabilistic streamline q-ball tractography using the residual bootstrap. Neuroimage 39, 215–222 10.1016/j.neuroimage.2007.08.021 17911030

[B14] Bilgic B. Setsompop K. Cohen-Adad J. Yendiki A. Wald L. Adalsteinsson E. (2012). Accelerated diffusion spectrum imaging with compressed sensing using adaptive dictionaries. Magn. Reson. Med. 68, 1747–1754 10.1002/mrm.24505 23008145 PMC3504650

[B15] Callaghan P. Eccles C. Xia Y. (1988). Rapid communication: NMR microscopy of dynamic displacements: k-space and q-space imaging. J. Phys. E Sci. Instrum. 21, 820–822 10.1088/0022-3735/21/8/017

[B16] Callaghan P. T. (1991). Principles of Nuclear Magnetic Resonance Microscopy. Oxford: Oxford University Press

[B17] Canales-Rodréiguez E. J. Iturria-Medina Y. Aleman-Gomez Y. Melie-Garcia L. (2010). Deconvolution in diffusion spectrum imaging. Neuroimage 50, 136–149 10.1016/j.neuroimage.2009.11.066 19962440

[B18] Caruyer E. Lenglet C. Sapiro G. Deriche R. (2013). Design of multishell sampling schemes with uniform coverage in diffusion MRI. Magn. Reson. Med. 69, 1534–1540 10.1002/mrm.24736 23625329 PMC5381389

[B19] Chamberland M. Descoteaux M. (2013). Explore the brain white matter networks in real-time: multi-sticks fiber tracking, in The International Society for Magnetic Resonance in Medicine (Sherbrooke).

[B20] Chang L.-C. Jones D. K. Pierpaoli C. (2005). RESTORE: robust estimation of tensors by outlier rejection. Magn. Reson. Med. 53, 1088–1095 10.1002/mrm.20426 15844157

[B21] Chang L.-C. Walker L. Pierpaoli C. (2012). Informed RESTORE: a method for robust estimation of diffusion tensor from low redundancy datasets in the presence of physiological noise artifacts. Magn. Reson. Med. 68, 1654–1663 10.1002/mrm.24173 22287298 PMC3615560

[B22] Chung S. Lu Y. Henry R. G. (2006). Comparison of bootstrap approaches for estimation of uncertainties of DTI parameters. Neuroimage 33, 531–541 10.1016/j.neuroimage.2006.07.001 16938472

[B23] Clayden J. D. Maniega S. M. Storkey A. J. King M. D. Bastin M. E. Clark C. A. (2011). TractoR: magnetic resonance imaging and tractography with R. J. Stat. Softw. 44, 1–18

[B24] Conturo T. Lori N. Cull T. Akbudak E. Snyder A. Shimony J. (1999). Tracking neuronal fiber pathways in the living human brain. Proc. Natl. Acad. Sci. U.S.A. 96, 10422–10427 10.1073/pnas.96.18.10422 10468624 PMC17904

[B25] Cook P. Bai Y. Nedjati-Gilani S. Seunarine K. Hall M. Parker G. (2006). Camino: open-source diffusion-MRI reconstruction and processing. Int. Soc. Magn. Reson. Med. 14, 2759

[B26] Côté M.-A. Girard G. Boré A. Garyfallidis E. Houde J.-C. Descoteaux M. (2013). Tractometer: towards validation of tractography pipelines. Med. Image Anal. 17, 844–857 10.1016/j.media.2013.03.009 23706753

[B27] Cox R. W. (2012). AFNI: what a long strange trip it's been. Neuroimage 62, 743–747 10.1016/j.neuroimage.2011.08.056 21889996 PMC3246532

[B28] Daducci A. Gerhard S. Griffa A. Lemkaddem A. Cammoun L. Gigandet X. (2012). The connectome mapper: an open-source processing pipeline to map connectomes with MRI. PLoS ONE 7:e48121 10.1371/journal.pone.0048121 23272041 PMC3525592

[B29] Descoteaux M. (2008). “High Angular Resolution Diffusion MRI: From Local Estimation to Segmentation and Tractography.” Ph.D. thesis, Université de Nice-Sophia Antipolis

[B30] Descoteaux M. Angelino E. Fitzgibbons S. Deriche R. (2006). Apparent diffusion coefficients from high angular resolution diffusion imaging: estimation and applications. Magn. Reson. Med. 56, 395–410 10.1002/mrm.20948 16802316

[B31] Descoteaux M. Angelino E. Fitzgibbons S. Deriche R. (2007). Regularized, fast, and robust analytical q-ball imaging. Magn. Reson. Med. 58, 497–510 10.1002/mrm.21277 17763358

[B32] Descoteaux M. Deriche R. Bihan D. L. Mangin J.-F. Poupon C. (2011). Multiple q-shell diffusion propagator imaging. Med. Image Anal. 15, 603–621 10.1016/j.media.2010.07.001 20685153

[B33] Descoteaux M. Deriche R. Knösche T. R. Anwander A. (2009). Deterministic and probabilistic tractography based on complex fibre orientation distributions. IEEE Trans. Med. Imaging 28, 269–286 10.1109/TMI.2008.2004424 19188114

[B34] Donoho D. L. (2010). An invitation to reproducible computational research. Biostatistics 11, 385–388 10.1093/biostatistics/kxq028 20538873

[B35] Ennis D. B. Kindlmann G. (2006). Orthogonal tensor invariants and the analysis of diffusion tensor magnetic resonance images. Magn. Reson. Med. 55, 136–146 10.1002/mrm.20741 16342267

[B36] Fischl B. (2012). Freesurfer. Neuroimage 62, 774–781 10.1016/j.neuroimage.2012.01.021 22248573 PMC3685476

[B37] Fortin D. Aubin-lemay C. Boré A. Girard G. Houde J.-C. Whittingstall K. (2012). Tractography in the study of the human brain: a neurosurgical perspective. Can. J. Neurol. Sci. 39, 747–756 23230612 10.1017/s0317167100015560

[B38] Frank L. R. (2001). Anisotropy in high angular resolution diffusion-weighted MRI. Magn. Reson. Med. 45, 935–939 10.1002/mrm.1125 11378869

[B39] Frank L. R. (2002). Characterization of anisotropy in high angular resolution diffusion-weighted MRI. Magn. Reson. Med. 47, 1083–1099 10.1002/mrm.10156 12111955

[B40] Fritzsche K. Neher P. Reicht I. van Bruggen T. Goch C. Reisert M. (2012). MITK diffusion imaging. Methods Inform. Med. 51, 441 10.3414/ME11-02-0031 23038239

[B41] Garyfallidis E. (2012). Towards an Accurate Brain Tractography. Ph.D. thesis, University of Cambridge, Cambridge

[B42] Garyfallidis E. Brett M. Amirbekian B. Nguyen C. Yeh F. Halchenko Y. (2011). Dipy - a novel software library for diffusion MR and tractography, in 17th Annual Meeting of the Organization for Human Brain Mapping (Cambridge).

[B43] Garyfallidis E. Brett M. Correia M. M. Williams G. B. Nimmo-Smith I. (2012). QuickBundles, a method for tractography simplification. Front. Neurosci. 6:175 10.3389/fnins.2012.00175 23248578 PMC3518823

[B44] Garyfallidis E. St-Jean S. Paquette M. Coupé P. Descoteaux M. (2013). Constrained spherical deconvolution on signal and ODF values, in ISBI HARDI reconstruction challenge 2013 (San Francisco, CA).

[B45] Goebel R. (2012). Brainvoyager past, present, future. Neuroimage 62, 748–756 10.1016/j.neuroimage.2012.01.083 22289803

[B46] Gorgolewski K. Burns C. Madison C. Clark D. Halchenko Y. Waskom M. (2011). Nipype: a flexible, lightweight and extensible neuroimaging data processing framework in python. Front. Neuroinform. 5:13 10.3389/fninf.2011.00013 21897815 PMC3159964

[B47] Gramfort A. Poupon C. Descoteaux M. (2013). Denoising and fast diffusion imaging with physically constrained sparse dictionary learning. Med. Image Anal. 18, 36-49 10.1016/j.media.2013.08.006 24084469

[B48] Halchenko Y. O. Hanke M. (2012). Open is not enough. letŠs take the next step: an integrated, community-driven computing platform for neuroscience. Front. Neuroinform. 6:22 10.3389/fninf.2012.00022 23055966 PMC3458431

[B49] Hess C. Mukherjee P. Han E. Xu D. Vigneron D. (2006). Q-ball reconstruction of multimodal fiber orientations using the spherical harmonic basis. Magn. Reson. Med. 56, 104–117 10.1002/mrm.20931 16755539

[B50] Jeurissen B. Leemans A. Jones D. K. Tournier J.-D. Sijbers J. (2011). Probabilistic fiber tracking using the residual bootstrap with constrained spherical deconvolution. Hum. Brain Mapp. 32, 461–479 10.1002/hbm.21032 21319270 PMC6869960

[B51] Jones D. (ed.). (2010). Diffusion MRI: Theory, Methods And Applications. Oxford, NY: Oxford University Press 10.1093/med/9780195369779.001.0001

[B52] Jones D. K. Horsfield M. A. Simmons A. (1999). Optimal strategies for measuring diffusion in anisotropic systems by magnetic resonance imaging. Magn. Reson. Med. 42, 515–525 10.1002/(SICI)1522-2594(199909)42:3<515::AID-MRM14>3.3.CO;2-H 10467296

[B53] Jones D. K. Knösche T. R. Turner R. (2013). White matter integrity, fiber count, and other fallacies: the do's and don'ts of diffusion MRI. Neuroimage 73, 239–254 10.1016/j.neuroimage.2012.06.081 22846632

[B54] Koay C. G. Chang L.-C. Carew J. D. Pierpaoli C. Basser P. J. (2006). A unifying theoretical and algorithmic framework for least squares methods of estimation in diffusion tensor imaging. J. Magn. Reson. 182, 115–125 10.1016/j.jmr.2006.06.020 16828568

[B55] LeBihan D. Breton E. (1985). Imagerie de diffusion *in vivo* par résonance magnétique nucléaire. C. R. Acad. Sci. Paris 301, 1109–1112

[B56] Leemans A. Jeurissen B. Sijbers J. Jones D. (2009). ExploreDTI: a graphical toolbox for processing, analyzing, and visualizing diffusion MR data. Proc. Int. Soc. Magn. Reson. Med. 17, 3537

[B57] Lenglet C. Rousson M. Deriche R. Faugeras O. (2006). Statistics on the manifold of multivariate normal distributions: theory and application to diffusion tensor mri processing. J. Math. Imaging Vis. 25, 423–444 10.1007/s10851-006-6897-z

[B58] Maas L. C. Mukherjee P. (2005). Diffusion MRI: overview and clinical applications in neuroradiology. Appl. Radiol. 34, 44–60 16649199

[B59] Maximilien E. M. Williams L. (2013). Assessing test-driven development at IBM, in Proceedings of the 25th International Conference on Software Engineering, 2003, Vol. 6 (Washington, DC). 10.1109/ICSE.2003.1201238

[B60] McKinney W. (2012). Python for Data Analysis. Sebastopol, CA: O'Reilly Media, Incorporated

[B61] Menzel M. Tan E. Khare K. Sperl J. King K. Tao X. (2011). Accelerated diffusion spectrum imaging in the human brain using compressed sensing. Magn. Reson. Med. 66, 1226–1233 10.1002/mrm.23064 22012686

[B62] Merboldt K. Hanicke W. Frahm J. (1985). Self-diffusion NMR imaging using stimulated echoes. J. Magn. Reson. 64, 479–486

[B63] Mori S. Crain B. J. Chacko V. P. Van Zijl P. C. M. (1999). Three-dimensional tracking of axonal projections in the brain by magnetic resonance imaging. Annal. Neurol. 45, 265–269 10.1002/1531-8249(199902)45:2<265::AID-ANA21>3.0.CO;2-3 9989633

[B64] Mori S. Wakana S. Nagae-Poetscher L. M. van Zijl P. C. M. (2005). MRI Atlas of Human White Matter. Amsterdam: Elsevier

[B65] Morris D. M. Embleton K. V. Parker G. J. (2008). Probabilistic fibre tracking: differentiation of connections from chance events. Neuroimage 42, 1329–1339 10.1016/j.neuroimage.2008.06.012 18619548

[B66] Numano T. Homma K. Iwasaki N. Hyodo K. Nitta N. Hirose T. (2006). *In vivo* isotropic 3d diffusion tensor mapping of the rat brain using diffusion-weighted 3d mp-rage MRI. Magn. Reson. Imaging 24, 287–293 10.1016/j.mri.2005.12.011 16563958

[B67] Otsu N. (1979). Threshold selection method from gray-level histograms. IEEE Trans. Syst. Man Cybern. SMC-9, 62–66 10.1109/TSMC.1979.4310076

[B68] Özarslan E. Koay C. G. Shepherd T. M. Komlosh M. E. İrfanoğlu M. O. Pierpaoli C. (2013). Mean apparent propagator (map) mri: a novel diffusion imaging method for mapping tissue microstructure. Neuroimage 78, 16–32 10.1016/j.neuroimage.2013.04.016 23587694 PMC4059870

[B69] Pajevic S. Pierpaoli C. (1999). Color schemes to represent the orientation of anisotropic tissues from diffusion tensor data: application to white matter fiber tract mapping in the human brain. Magn. Reson. Med. 42, 526–540 10.1002/(SICI)1522-2594(199909)42:3<526::AID-MRM15>3.3.CO;2-A 10467297

[B70] Pérez F. Granger B. E. (2007). Ipython: a system for interactive scientific computing. Comput. Sci. Eng. 9, 21–29 10.1109/MCSE.2007.53

[B71] Pérez F. Granger B. E. Hunter J. D. (2011). Python: an ecosystem for scientific computing. Comput. Sci. Eng. 13, 13–21 10.1109/MCSE.2010.119

[B72] Pieper S. Lorensen B. Schroeder W. Kikinis R. (2006). The NA-MIC Kit: ITK, VTK, pipelines, grids and 3D slicer as an open platform for the medical image computing community, in 3rd IEEE International Symposium on Biomedical Imaging: Nano to Macro, 2006 (Arlington, VA: IEEE), 698–701 10.1109/ISBI.2006.1625012

[B73] Pierpaoli C. Jezzard P. Basser P. Barnett A. Chiro G. D. (1996). Diffusion Tensor MR imaging of human brain. Radiology 201, 637–648 8939209 10.1148/radiology.201.3.8939209

[B74] Schroeder W. J. Avila L. S. Martin K. M. Hoffman W. A. Law C. C. (2001). The Visualization Toolkit-User's Guide. Clifton Park, NY: Kitware, Inc

[B75] Setsompop K. Cohen-Adad J. Gagoski B. Raij T. Yendiki A. Keil B. (2012). Improving diffusion mri using simultaneous multi-slice echo planar imaging. Neuroimage 63, 569–580 10.1016/j.neuroimage.2012.06.033 22732564 PMC3429710

[B76] Sherbondy A. Akers D. Mackenzie R. Dougherty R. Wandell B. (2005). Exploring connectivity of the brain's white matter with dynamic queries. IEEE Trans. Vis. Comput. Graph. 11, 419–430 10.1109/TVCG.2005.59 16138552

[B77] Smith S. M. Jenkinson M. Woolrich M. W. Beckmann C. F. Behrens T. Johansen-Berg H. (2004). Advances in functional and structural MR image analysis and implementation as FSL. Neuroimage 23, S208–S219 10.1016/j.neuroimage.2004.07.051 15501092

[B78] Song S.-K. Sun S.-W. Ramsbottom M. J. Chang C. Russell J. Cross A. H. (2002). Dysmyelination revealed through MRI as increased radial (but unchanged axial) diffusion of water. Neuroimage 17, 1429–1436 10.1006/nimg.2002.1267 12414282

[B79] Sporns O. Tononi G. Kötter R. (2005). The human connectome: a structural description of the human brain. PLoS Comput. Biol. 1:e42 10.1371/journal.pcbi.0010042 16201007 PMC1239902

[B80] Taylor D. Bushell M. (1985). The spatial mapping of translational diffusion coefficients by the nmr imaging technique. Phys. Med. Biol. 30, 345–349 10.1088/0031-9155/30/4/009 4001161

[B81] Thomason M. E. Thompson P. M. (2011). Diffusion imaging, white matter, and psychopathology. Annu. Rev. Clin. Psychol. 7, 63–85 10.1146/annurev-clinpsy-032210-104507 21219189

[B82] Tournier J.-D. Calamante F. Connelly A. (2007). Robust determination of the fibre orientation distribution in diffusion mri: non-negativity constrained super-resolved spherical deconvolution. Neuroimage 35, 1459–1472 10.1016/j.neuroimage.2007.02.016 17379540

[B83] Tournier J.-D. Calamante F. Connelly A. (2012). MRtrix: diffusion tractography in crossing fiber regions. Int. J. Imaging Syst. Technol. 22, 53–66 10.1002/ima.2200521182970

[B84] Tournier J.-D. Calamante F. Gadian D. Connelly A. (2004). Direct estimation of the fiber orientation density function from diffusion-weighted MRI data using spherical deconvolution. Neuroimage 23, 1176–1185 10.1016/j.neuroimage.2004.07.037 15528117

[B85] Toussaint N. Souplet J. Fillard P. (2007). MedINRIA: medical image navigation and research tool by INRIA, in MICCAI'07 Workshop on Interaction in medical image analysis and visualization (Brisbane, Australia).

[B86] Tristan-Vega A. Westin C.-F. Aja-Fernandez S. (2009). Estimation of fiber orientation probability density functions in high angular resolution diffusion imaging. Neuroimage 47, 638–650 10.1016/j.neuroimage.2009.04.049 19393321

[B87] Tuch D. (2004). Q-ball imaging. Magn. Reson. Med. 52, 1358–1372 10.1002/mrm.20279 15562495

[B88] Tuch D. Reese T. Wiegell M. Makris N. Belliveau J. Wedeen V. (2002). High angular resolution diffusion imaging reveals intravoxel white matter fiber heterogeneity. Magn. Reson. Med. 48, 577–582 10.1002/mrm.10268 12353272

[B89] Tuch D. S. (2002). Diffusion MRI of Complex Tissue Structure. Ph.D. thesis, Harvard University and Massachusetts Institute of Technology

[B90] Vaillancourt O. Boré A. Girard G. Descoteaux M. (2011). A fiber navigator for neurosurgical planning, in Organization for Human Brain Mapping (Sherbrooke, QC).

[B91] van der Walt S. Colbert S. C. Varoquaux G. (2011). The NumPy array: a structure for efficient numerical computation. Comput. Sci. Eng. 13, 22–30 10.1109/MCSE.2011.37

[B92] Van Essen D. C. Smith S. M. Barch D. M. Behrens T. E. J. Yacoub E. Ugurbil K. (2013). The WU-Minn human connectome project: an overview. Neuroimage 80, 62–79 10.1016/j.neuroimage.2013.05.041 23684880 PMC3724347

[B93] Wandell B. A. Yeatman J. D. (2013). Biological development of reading circuits. Curr. Opin. Neurobiol. 23, 261–268 10.1016/j.conb.2012.12.005 23312307 PMC3622751

[B94] Wang R. Benner T. Sorensen A. Wedeen V. (2007). Diffusion toolkit: a software package for diffusion imaging data processing and tractography. Proc. Int. Soc. Magn. Reson. Med. 15, 3720

[B95] Wedeen V. J. Hagmann P. Tseng W.-Y. I. Reese T. G. Weisskoff R. M. (2005). Mapping complex tissue architecture with diffusion spectrum magnetic resonance imaging. Magn. Reson. Med. 54, 1377–1386 10.1002/mrm.20642 16247738

[B96] Westin C.-F. Peled S. Gudbjartsson H. Kikinis R. Jolesz F. A. (1997). Geometrical diffusion measures for MRI from tensor basis analysis. Proc. ISMRM 97, 1742

[B97] Yeatman J. D. Dougherty R. F. Myall N. J. Wandell B. A. Feldman H. M. (2012). Tract profiles of white matter properties: automating fiber-tract quantification. PLoS ONE 7:e49790 10.1371/journal.pone.0049790 23166771 PMC3498174

[B98] Yeh F. Wedeen V. Tseng W. (2010). Generalized Q-sampling imaging. IEEE Trans. Med. Imaging 29, 1626–1635 10.1109/TMI.2010.2045126 20304721

